# Beyond integration: towards benchmarks for developmental potential in human stem cell-derived embryo models

**DOI:** 10.1093/humupd/dmaf033

**Published:** 2026-01-05

**Authors:** Dorian G Luijkx, Leila Ashtar, Nienke de Graeff, Edith Coonen, Stefan Giselbrecht, Guido M W R de Wert, Erik J Vrij, Rhiannon Grant, Ana M Pereira Daoud

**Affiliations:** Department for Cell Biology-Inspired Tissue Engineering, MERLN Institute for Technology-Inspired Regenerative Medicine, Maastricht University, Maastricht, The Netherlands; Department for Cell Biology-Inspired Tissue Engineering, MERLN Institute for Technology-Inspired Regenerative Medicine, Maastricht University, Maastricht, The Netherlands; Department of Medical Ethics and Health Law, Leiden University Medical Center, Leiden, The Netherlands; The Novo Nordisk Foundation Center for Stem Cell Medicine (reNEW), Leiden Node, Leiden, The Netherlands; Department of Clinical Genetics, Maastricht University Medical Center, Maastricht, The Netherlands; Department of Obstetrics & Gynecology, GROW Research Institute for Oncology and Reproduction, Maastricht University, Maastricht, The Netherlands; Department of Reproductive Medicine, Maastricht University Medical Center+, Maastricht, The Netherlands; Department for Cell Biology-Inspired Tissue Engineering, MERLN Institute for Technology-Inspired Regenerative Medicine, Maastricht University, Maastricht, The Netherlands; Department of Obstetrics & Gynecology, GROW Research Institute for Oncology and Reproduction, Maastricht University, Maastricht, The Netherlands; Department of Health, Ethics and Society, Maastricht University, Maastricht, The Netherlands; Care and Public Health Research Institute (CAPHRI), Maastricht University, Maastricht, The Netherlands; Department for Cell Biology-Inspired Tissue Engineering, MERLN Institute for Technology-Inspired Regenerative Medicine, Maastricht University, Maastricht, The Netherlands; Department of Obstetrics & Gynecology, GROW Research Institute for Oncology and Reproduction, Maastricht University, Maastricht, The Netherlands; Institute for Bioengineering, School of Engineering, University of Edinburgh, Edinburgh, UK; Department of Medical Ethics and Health Law, Leiden University Medical Center, Leiden, The Netherlands

**Keywords:** human stem cell-based embryo models, benchmarks, integration, developmental potential, governance, science, ethics

## Abstract

**BACKGROUND:**

Stem cell-based embryo models (SCBEMs) are clusters of pluripotent stem cells that can mimic morphological and functional aspects of early human embryos to different degrees. When cultured from human cells, SCBEMs offer technically scalable and amenable tools that can help refine, reduce, and, in the future, perhaps replace the use of animals and human embryos in fundamental and clinical research. These advantages propelled the development of SCBEMs, and several distinct types have been generated over the past decade, including gastruloids, axioloids, blastoids, and post-implantation-like embryoids. For purposes of governance, advisory reports distinguish between SCBEMs based on their presumed capacity to continuously undergo organized human development—referred to here as developmental potential. However, since functionally testing this potential by transferring human SCBEMs to a uterus would be unethical and is recommended to be prohibited, scientists lack clear or consistent ways to assess it.

**OBJECTIVE AND RATIONALE:**

This narrative review aims to tackle the question of how to assess developmental potential in SCBEMs by clarifying the different ways in which it can be and is being conceptualized. We achieve this by synthesizing insights from governance, science, and ethics. First, we examine how developmental potential is described in contemporary governance frameworks, and which aspects are emphasized. Next, we discuss biological markers for developmental potential and show how their scientific basis (in embryos, let alone SCBEMs) remains poorly understood. Then, we explore how the aspects considered relevant for assessments of developmental potential in governance and science may pre-emptively hinge on underlying conceptual interpretations and lead to differing normative implications.

**SEARCH METHODS:**

This narrative review combines insights from both the academic and grey literature on the (ethics of) embryo models. Original and review articles were selected from PubMed and Biorxiv with the main focus on articles published since 2015. Search terms included: embryo quality, *in vitro* fertilization, Gardner system, blastoid, gastruloid, embryo research, potentiality argument, developmental potential, transcriptomics, epigenetics, embryo metabolism, and related terms. Additional sources were identified through snowballing. This work focuses predominantly on human SCBEMs, but references to animal models are made.

**OUTCOMES:**

Comparison of the descriptions currently recommended for governance suggests at least three criteria that are used to assess developmental potential in SCBEMs: composition, organization, and interaction. Scientifically, developmental potential is multifaceted and only partly characterized, making it necessary to measure a broader range of aspects, using human embryos as benchmarks when possible. Since the range and significance of these aspects can be shaped by underlying accounts of developmental potential, contemporary advisory reports are examined to explore if and how they connote interpretations of developmental potential as possibility, probability, and predisposition.

**WIDER IMPLICATIONS:**

Categorization of the regulatory and scientific criteria currently used to assess developmental potential shows that they are underpinned by distinct interpretations of the concept, revealing tensions and questions for further inquiry. By synthesizing insights from governance, science, and ethics, this review thus aims to contribute to the responsible advancement of the SCBEM field and to support its coherent and transparent governance.

**REGISTRATION NUMBER:**

N/A.

## Graphical abstract

**Figure:**
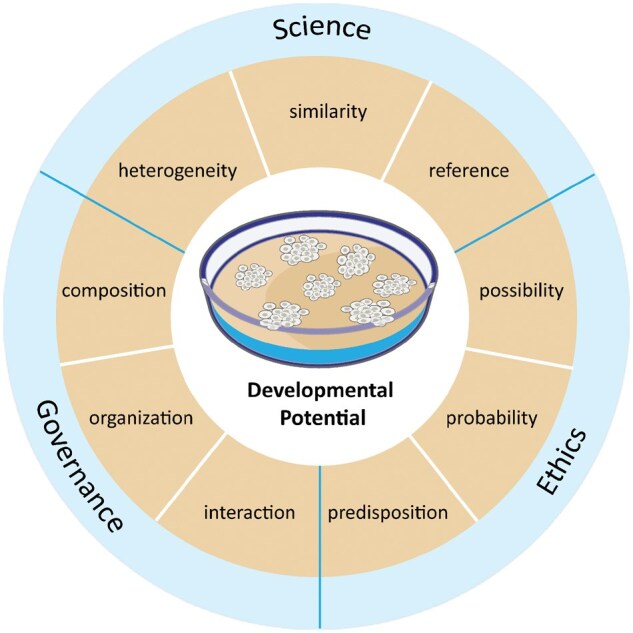
**Evaluating developmental potential in human stem cell-based embryo models from the perspective of governance, science, and ethics.** Petri dish image and generic cell images were used from NIAID Visual & Medical Arts (10/7/2024). NIAID NIH BIOART URL: bioart.niaid.nih.gov/bioart/172 and bioart.niaid.nih.gov/bioart/404

## Introduction

**Figure 1.**
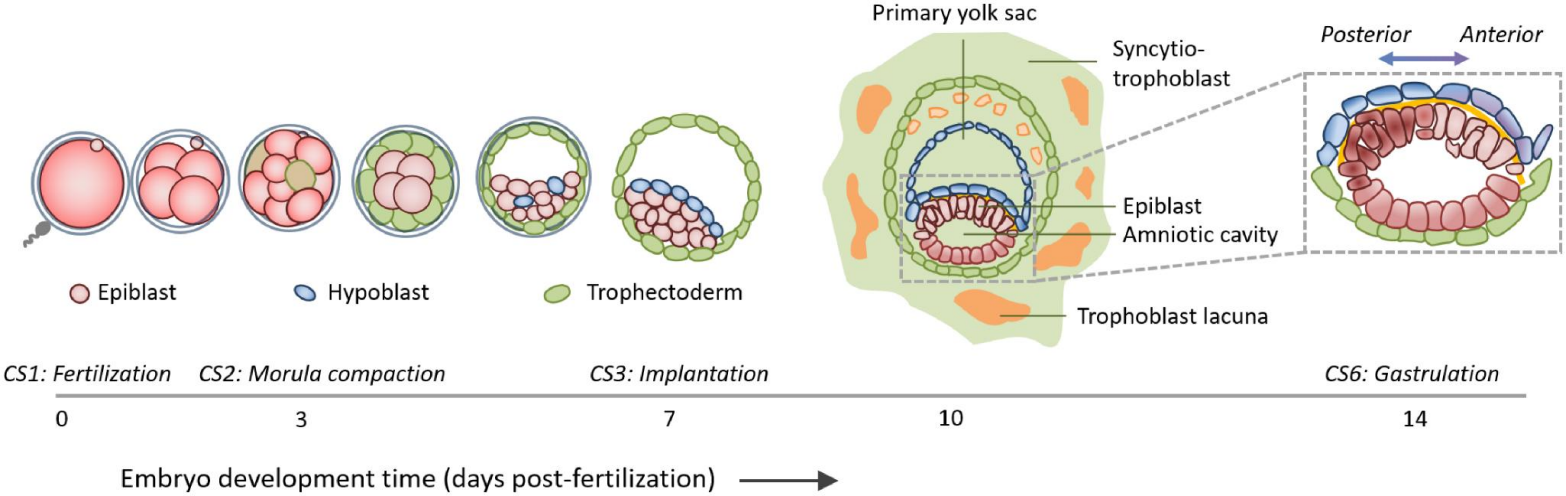
**Human embryo development from fertilization to Day 14.** Shown is the progression from zygote to implanted embryo and its initial development. Key stages include cleavage, blastocyst formation with segregation of epiblast (red), hypoblast (blue), and trophectoderm (green), followed by implantation, pro-amniotic cavity formation, and axis specification. The inset depicts the bilaminar disc at Day 14.

Stem cell-based embryo models (SCBEMs) are a heterogeneous group of entities generated from pluripotent stem cells (PSCs) that recapitulate morphological and functional features of (early) human embryos *in vitro* (Fig. 1; Supplementary Data File S1). SCBEMs provide unprecedented avenues for researchers to gain fundamental insights into human development, with potential future clinical applications (Posfai *et al.*, 2021; Rossant and Tam, 2021). Compared to mammalian embryos, SCBEMs are scalable and more amenable to scientific manipulation. In addition, SCBEMs may provide ways to bypass some of the normative drawbacks of animal and human embryo research (Pereira Daoud *et al.*, 2020; Nicolas *et al.*, 2021; Iltis *et al.*, 2023; Marei, 2025).

Within the past decade, the field has advanced rapidly and produced several distinct types of (human and non-human) SCBEMs (Shankar *et al.*, 2021). Some of them model specific developmental processes, like gastrulation, axis development, or somitogenesis. Present-day examples include gastruloids (van den Brink *et al.*, 2014; Beccari *et al.*, 2018; Moris *et al.*, 2020), axioloids (Yamanaka *et al.*, 2023), and extra-embryoids (Pedroza *et al.*, 2023), respectively. Other models attempt to mimic the entire conceptus, including extraembryonic tissues. Examples include blastoids (Rivron *et al.*, 2018; Fan *et al.*, 2021; Liu *et al.*, 2021; Yanagida *et al.*, 2021; Kagawa *et al.*, 2022; Karvas *et al.*, 2023; Yu *et al.*, 2023) and early post-implantation SCBEMs (e.g. gastrulating embryo-like structures (Sozen *et al.*, 2018), sEmbryos (Tarazi *et al.*, 2022), SEMs (Oldak *et al.*, 2023), and embryoids (Weatherbee *et al.*, 2023)).

Several advisory reports have recommended that governance frameworks distinguish between human SCBEMs based on whether they possess the (embryonic and extra-embryonic) cells required to continuously undergo organized development. This led to the emergence of conceptual dichotomies like ‘integrated vs non-integrated’ (ISSCR, 2021; Lovell-Badge *et al.*, 2021; Writing Group of the ESHRE Ethics Committee *et al.*, 2024), or ‘complete vs incomplete’ (Dondorp *et al.*, 2021) SCBEMs, among others. For consistency, and in view of their widespread use, this manuscript will use the terms ‘integrated’ and ‘non-integrated’ to distinguish between SCBEMs that model the entire conceptus and those that do not, respectively. More recent advisory reports have recommended abandoning conceptual dichotomies because they cannot meaningfully address ‘the complexity and heterogeneity of more recent (post-2021) human SCBEM technologies’ (Clark et al., 2025), which have been shown to be able to develop intricate structures even in the absence of cell types that were considered essential in past dichotomies.

Despite these differences, the contemporary focus on the ability of developing to more advanced stages in an organized manner underscores a shared underlying assumption: SCBEMs that (may) possess a capacity for organized human development deserve greater normative consideration than those that lack it. In the scientific literature, this has led to calls for systematic investigation of SCBEMs and the biological features that can help scientists determine SCBEMs’ capacity for continuously organized development (Martinez Arias *et al.*, 2024). In the ethics literature, it links with the historical role of the so-called Argument From Potential (AFP), which is taken to reflect the commonly held view that some (clusters of) human cells matter more than others because of what they can become: a human person like us, rather than one of its parts. However, since testing this by transferring human SCBEMs to a uterus would be unethical and is recommended to be prohibited, scientists and regulators lack clear or consistent ways to assess it (Villalba *et al.*, 2024).

## Methods

This review draws on academic and grey literature—identified through PubMed and bioRxiv and subsequent snowballing, with a focus on publications since 2015—to address the question of how to assess developmental potential in SCBEMs. This question arises from a governance, scientific, and ethical perspective, discussed as such in the background sections: (i) expert recommendations emphasize different aspects when describing developmental potential, (ii) the scientific basis of developmental potential (in embryos, let alone SCBEMs) remains poorly understood, and (iii) what aspects are treated as relevant evidence often depends on pre-existing conceptual accounts of developmental potential—as possibility, probability, or predisposition—with varying normative implications.

The discussion tackles the question of how to more clearly conceptualize and assess developmental potential in the context of human SCBEM. First, we identify common criteria across expert recommendations. Then, we analyse how scientific assays used in IVF contexts might be adapted to assess developmental potential in SCBEMs. Finally, we examine whether and how expert recommendations connote (elements of) the different accounts of developmental potential. By integrating insights from governance, science, and ethics, our discussion aims to clarify how developmental potential can be conceptualized and assessed. In doing so, it supports coherent and transparent governance of SCBEM research.

## Background

The following sections examine how the concept of developmental potential functions across the governance, science, and ethics of SCBEMs. We begin by reviewing if and how advisory reports connote the concept when differentiating between different types of SCBEMs. Next, we assess if and how scientists may be able to measure and benchmark proxies of this potential, particularly in relation to embryos derived from IVF. Finally, we explore how developmental potential has been interpreted in the ethics literature and how specific interpretations bear on questions of moral status.

### Developmental potential in governance

Over the past few years, several efforts have been made to develop guidelines for research involving human SCBEMs. Policy recommendations have been set out by, for example, advisors in the Netherlands (Dondorp *et al.*, 2021; HCN, 2023), Australia (NHMRC, 2022), France (Bruno *et al.*, 2023), Sweden (SMER, 2023, 2024), and the UK (HFEA, 2023; NCOB, 2024; Sturmey *et al.*, 2024), as well as expert committees of international professional groups, such as the International Society for Stem Cell Research (ISSCR) (ISSCR, 2021, 2025) and the European Society of Human Reproduction and Embryology (ESHRE) (Writing Group of the ESHRE Ethics Committee *et al.*, 2024). Notably, however, expert recommendations diverge on both conceptual and normative levels.

Conceptually, some groups recommend using binary language to distinguish between types of SCBEMs (Dondorp *et al.*, 2021; NHMRC, 2022; HCN, 2023; SMER, 2023, 2024; Writing Group of the ESHRE Ethics Committee *et al.*, 2024), while others do not (NCOB, 2024; Sturmey *et al.*, 2024; ISSCR, 2025) (Table 1). Binary language gained prominence following the 2021 ISSCR Guidelines (ISSCR, 2021; Lovell-Badge *et al.*, 2021; Fabbri *et al.*, 2023), which introduced a distinction between integrated and non-integrated SCBEMs based on the premise that only the former contained the relevant cells to ‘potentially achieve the complexity by which they might realistically undergo further integrated development if cultured for additional time in appropriate conditions or, theoretically, if transferred to a uterus’ (Lovell-Badge *et al.*, 2021). Binary distinctions were adopted as simplified proxies for articulating differences in the presumed ‘developmental potential of the entity: can the entity, when placed in the right conditions, develop into a human being?’ (Writing Group of the ESHRE Ethics Committee *et al.*, 2024). However, they were criticized almost as quickly as celebrated, particularly after reports showing that some SCBEMs could undergo complex organized development even without cell types previously deemed necessary—a development that prompted recent expert recommendations (NCOB, 2024; Sturmey *et al.*, 2024), including the ISSCR’s (ISSCR, 2025), to abandon them.

**Table 1.** Chronological overview of advisory efforts to describe (specific types of) SCBEMs.

**Jurisdiction** (Reference)	Classification	Hyper-nym(s)	Hypo-nym(s)	Description(s)
**The Netherlands** (Dondorp *et al.*, 2021)	Binary	Embryo-like structures	Complete	SCBMS ‘in which the aim is to mimic the organized development of an intact human embryo’ (p. 196).
			Incomplete	SCBEMs in which the aim is not to mimic the organized development of an intact human embryo (cf. p. 86).
**International** (ISSCR, 2021)	Binary	Stem cell-based embryo models	Integrated	SCBEMs that ‘contain the relevant embryonic and extra-embryonic structures and could potentially achieve the complexity where they might realistically manifest the ability to undergo further integrated development if cultured for additional time *in vitro*’ (p. 64).
			Non-integrated	SCBEMs that ‘experimentally recapitulate some, but not all aspects of the per-implantation embryo, for example differentiation of the embryonic sac or embryonic disc in the absence of extraembryonic cells’ (p. 64).
**Australia** (NHMRC, 2022)	Binary	Embryo models	Intact (Embryos)	SCBEMs that meet the definition of the human embryo, which defines it as ‘human cells with a human nuclear genome or altered human nuclear genome (including human stem cells, induced pluripotent stem cells, somatic cells, gametes, etc)’; and (…) ‘The entity has the potential to develop up to, or beyond, the stage at which the primitive streak appears. An embryo model meets this definition even if it does not: follow the normal embryonic development process, or undergo epithelial-to-mesenchymal transition (EMT) and/or form a primitive streak, or pass through the stage of gastrulation’; and (…) ‘The entity has arisen from fertilisation or any other process that initiates organised development of a biological entity. It has met this requirement if: (i) There is evidence of developmental attributes contributing to spatial and/or temporal organisation within the entity; and (ii) It is an intact conceptus, which has the potential to develop the full complement of parts of a normally developing embryo (including embryonic cells that give rise to all the cells in a human body and extraembryonic tissues such as the amnion and placenta)’.
			Non-intact	SCBEMs that meet ‘some parts of the definition of a human embryo. However, [do] not fully demonstrate ‘organised development of a biological entity’ on the basis that it is not an intact conceptus with the potential to develop the full complement of parts of a normally developing embryo (including embryonic cells that give rise to all the cells in a human body and extraembryonic tissues such as the amnion and placenta)’.
**Sweden** (SMER, 2023)	Binary	Stem cell-based embryo models	Integrated	SCBEMs that ‘aim to represent embryo development as a whole. They include extra-embryonic cells and can achieve a level of complexity where they are capable of undergoing further development’ (p. 6).
			Non-integrated	SCBEMs that ‘mimic some, but not all, aspects of the early embryo. As a rule, they lack extra-embryonic cells which do not give rise to a foetus but are necessary for the embryo’s continued development. Non-integrated embryo models are therefore not capable of further development’ (p. 6).
**The Netherlands** (HCN, 2023)	Binary	Embryo-like structures	Intact (Non-conventional embryos)	SCBEMs that ‘mimic all aspects of intact embryos’ (p. 44) and are described as ‘… biological entities with a (largely) human genome, which have indeed arisen in a different way than through fertilization, but which do possess all the necessary cell types to be able to go through the normal embryonic stages as a whole. These intact [SCBEMs] look just like classical embryos under the microscope and behave—at least up to 14 days—the same as well’ (p. 45).
			Non-intact	SCBEMs that ‘can contain all types of embryonic cells, but in any case do not contain all the extra-embryonic tissues that are supposed to be present in the developmental stage that the [SCBEM] corresponds to, but only one organ or organ system’ (p. 44).
**France** (Bruno *et al.*, 2023)	Binary	Embryoids	Integrated	SCBEMs that ‘can reconstitute the entire embryo and at least part of the different extraembryonic appendages (trophoblast and primitive endoderm). Therefore, at the current state of knowledge, it is assumed that these models, after improvement of the protocols, could acquire the ability to form a fetus or even a newborn’ (p. 2).
			Non-integrated	SCBEMs that ‘will never be able to form a complete embryo, even if protocols are improved, because they lack certain embryonic and/or extraembryonic tissues’ (p. 2).
**UK** (UK Parliament Post, 2024)	Binary	Stem cell-based embryo models	Integrated	SCBEMs that ‘model the developmental processes of the embryo along with the extra-embryonic tissue. They are thought to have the possibility of acquiring the potential to develop into a foetus, if conditions and modelling improves’ (p. 4).
			Non-integrated	SCBEMs that ‘seek to reproduce a specific part or developmental process of the developing embryo. They do not include extra-embryonic cells and are therefore thought to lack the potential to develop into a foetus’ (p. 3).
**UK** (Sturmey *et al.*, 2024)	Gradual	Stem cell-based embryo models	N/A	For this expert group, ‘there is no distinction made in the Code between integrated and non-integrated SCBEMs. The code applies to all types of human SCBEMs irrespective of the starting material and degree of integration’ (p. 5).
**UK** (NCOB, 2024)	Gradual	Stem cell-based embryo models	N/A	For this expert group, ‘it is currently difficult to clearly define or categorize different types of SCBEM, both on a technical basis and according to what ethical or regulatory issues they may raise’ (p. 9).
**International** (Writing Group of the ESHRE Ethics Committee *et al.*, 2024)	Binary	Embryo-like structures	Integrated	SCBEMs that ‘contain all cell types required for the development of both the foetus and its supporting (extraembryonic) tissues’ (p. 2388).
			Non-integrated	SCBEMs that ‘are less complex and lack some (or several) tissue types’ (p. 2388).
**International** (ISSCR, 2025)	Gradual	Stem cell-based embryo models	N/A	SCBEMs ‘are the assembly, differentiation, aggregation, or re-association of cell populations in a manner that models or recapitulates key stages of embryonic development in 3D. These entities are designed to model specific phenotypic features and developmental processes of human embryos’ (p. 67). Past hyponyms were retired with 2025 ISSCR Guidelines update.

Normatively, expert recommendations interpret the implications of developmental potential for oversight and governance in different ways. Some argue that possessing such developmental potential collapses any relevant distinction between SCBEMs and embryos, making them morally, if not legally, equivalent. Australian (NHMRC, 2022), Dutch (Dondorp *et al.*, 2021; HCN, 2023), and Swedish (SMER, 2024) experts, for example, reason that SCBEMs with a developmental potential akin to human embryos should be regulated under the same governance framework as human embryos in research. In Australia, certain types of integrated SCBEMs are already considered to fulfil the statutory definition of ‘embryo’ (NHMRC, 2022), which includes human entities derived from fertilization or any other process capable of resulting in an intact conceptus that has the potential to develop up to, or beyond the stage at which the primitive streak appears. In the Netherlands and Sweden, the statutory definitions underpinning embryo research legislation are more difficult to apply to SCBEMs. In Sweden, the Genetic Integrity Act (2006) only defines the embryo implicitly, referring to research with human eggs that have been either fertilized or subject to SCNT. In the Netherlands, the embryo is explicitly defined as ‘a cell or cluster of cells with the potential to develop into a human being’ (Embryos Act, 2002), which cannot be tested ethically in humans and has yet to be demonstrated in non-human species. As such, SCBEMs presently fall outside the scope of Dutch and Swedish embryo legislation. While they consider this appropriate for contemporary models, Dutch and Swedish experts reason that regulators should either revise the statutory embryo definition or add specific provisions to include sophisticated versions of SCBEMs—which they further refer to as ‘complete’, ‘intact’, or ‘whole’—under national embryo laws (Dondorp *et al.*, 2021; HCN, 2023; SMER, 2024).

Other expert recommendations recognize that a developmental potential akin to human embryos would confer greater protection to the SCBEMs that possess it without taking that to mean that it would make them morally equivalent to human embryos. In the UK, for example, SCBEMs currently do not fall under the regulatory framework of embryos (HFEA, 2022; Jackson, 2024), and advisors disagree on whether future, more sophisticated versions should (Cave, 2025). According to the UK SCBEM Code of Practice, for instance, ‘were it ever considered, as a matter of best scientific judgement, that a SCBEM very likely has the potential to develop fully within a human host, it would no longer be appropriate to refer to it as a ‘model’; rather, it should then be viewed as an ‘embryo’, and would be governed as such’ (Sturmey *et al.*, 2024). The Nuffield Council on Bioethics, however, recommends instead that, ‘for *governance purposes*, the SCBEM is considered to be distinct from the embryo even if a time may come when, for *scientific purposes*, the two could become indistinguishable in terms of their capacity for onward development, as evidenced by the reliable detection of certain molecular and cellular proxies’ (NCOB, 2024). This aligns with the recommendations of the French Conseil d’orientation, according to which SCBEMs ‘cannot, in essence, be equivalent to embryos’ (Bruno *et al.*, 2023) due to their distinct origin (i.e. created from stem cells rather than gametes) and intentionality (i.e. created for research rather than reproduction). On this position, SCBEMs ‘are not embryos, but they model early embryonic development and enable scientific and medical advances. Therefore, they deserve a specific framework that should be more flexible than that for embryo research, but more stringent than that for research on traditional cell lines’ (Bruno *et al.*, 2023). Similarly, the Nuffield Council of Bioethics concludes, ‘notwithstanding the theoretical potential in the future for SCBEMs to pass a ‘Turing test’ of equivalence with embryos, our preferred solution is to govern embryos and SCBEMs separately. This is in recognition of the different origins and intentions associated with SCBEMs and, given the current framework for the regulation of embryos, the pragmatic advantages of separate governance which would facilitate targeted, proportionate, effective and bespoke oversight’ (NCOB, 2024).

Taken together, these recommendations point to an emerging consensus: experts agree that the developmental potential of SCBEMs plays a role in their governance, even if they disagree on how that potential should be conceptually and normatively evaluated. This raises the pressing question of how to determine and differentiate between the developmental potential of different models (Villalba *et al*., 2022).

### Developmental potential in science

Since assessing the developmental potential of human SCBEMs by transferring them to a uterus is considered unethical and recommended to be prohibited due to risks of serious harm (e.g. ISSCR, 2021, 2025; Bruno *et al.*, 2023; SMER, 2023; NCOB, 2024; Sturmey *et al.*, 2024), scientists need to make educated predictions about the developmental potential of SCBEMs through animal and *in vitro* comparisons.

To date, no SCBEMs—whether cultured from human or non-human cells—have demonstrated extended survival, full implantation competence, or continuously organized development *in vitro*, even if they possessed the full complement of cell lineages. Transfer of non-human models to *in vivo* uterine environments has not produced offspring yet, either. This indicates continuing technical limitations (Kagawa *et al.*, 2022; Yu *et al.*, 2023) and prevents contemporary models from being deemed equivalent to human embryos (Villalba *et al.*, 2024).

#### Diversity in SCBEMs

As Sozen *et al.* (2022) note, ‘*In vivo*, the acquisition of correct fate, form, and function are closely intertwined’ (50) but ‘*In vitro*, these can be disconnected’ (52). This interdependence complicates assessments of developmental potential, especially as *in vitro* development may be bioengineered to diverge from development *in vivo* by design.

Currently, most SCBEMs lack tissue types known to be present in the embryo at the developmental stage the model intends to mimic (Rossant and Tam, 2021). A prominent example is the so-called post-implantation amniotic sac embryoid (Shao *et al.*, 2017). This model recapitulates several events from the early human post-implantation stage, such as symmetry breaking of the epiblast compartment and the formation of the amniotic sac and posterior primitive streak, but lacks key components, such as placental development markers. Similarly restricted SCBEMs include gastruloids, axioloids, and extra-embryoids (Moris *et al.*, 2020; van den Brink and van Oudenaarden, 2021; Liu *et al.*, 2023; Pedroza *et al.*, 2023; Yamanaka *et al.*, 2023; Gribaudo *et al.*, 2024). Axioloids, for example, mimic axial elongation and somitogenesis and are morphologically and molecularly similar to the mesodermal compartment of the embryo. As such, axioloids can be used for in-depth analysis of the mechanisms underlying somitogenesis. However, since axioloids only consist of mesoderm-derived cell types, they cannot contain the progenitor cell types that give rise to the embryo proper (Yamanaka *et al.*, 2023). Gastruloids can contain all three germ layers of gastrulating embryos, but lack extraembryonic tissues. They represent a stage of embryo development where morphological complexity increases significantly and the foundations for all somatic tissues are laid (Moris *et al.*, 2020; van den Brink and van Oudenaarden, 2021), however, they are known to arrest after extended culture, indicating either a technical or an internal limitation that prevents them from undergoing continuously organized development.

Human extra-embryoids and peri-gastruloids contain embryonic and some extraembryonic tissues. Human extra-embryoids contain the early post-implantation embryonic compartment and the extraembryonic compartment that precedes the yolk sac, although not that which gives rise to placental tissues. Extra-embryoids are able to mimic spatial organization and specification of the epiblast towards the peri-gastrulation stage but, similarly to axioloids, only mesoderm-derived cells are detected (Pedroza *et al.*, 2023). Peri-gastruloids form extraembryonic tissues like the yolk and amniotic sac, as well as embryonic structures like the primitive streak. In addition, single-cell analysis of peri-gastruloids has shown the onset of organogenesis in a late stage of culture (Liu *et al.*, 2023). However, they lack trophectoderm/placental tissues.

Only a small number of SCBEMs currently comes close to resembling the whole conceptus: peri-implantation blastoids and post-implantation like embryoids (Liu *et al.*, 2021; Yanagida *et al.*, 2021; Kagawa *et al.*, 2022; Karvas *et al.*, 2023; Oldak *et al.*, 2023; Weatherbee *et al.*, 2023; Yu *et al.*, 2023; Luijkx *et al.*, 2024). These models contain embryonic and both extraembryonic lineages; all cell types present in the embryo at the respective stages that they are mimicking. Blastoids, for example, contain all the cell identities found in blastocysts, as confirmed by several independent groups and using various techniques, ranging from immunofluorescent staining of lineage markers to single-cell RNA sequencing (Fan *et al.*, 2021; Liu *et al.*, 2021; Yanagida *et al.*, 2021; Kagawa *et al.*, 2022; Yu *et al.*, 2023). Similarly, SEMs or embryoids contain the embryonic and extraembryonic lineages, and successfully recapitulate the organization and differentiation of these lineages into several post-implantation stage cell types (Oldak *et al.*, 2023; Weatherbee *et al.*, 2023).

#### Benchmarking SCBEMs

There are many quantifiable features that are important to assess scientific similarity to natural embryos (e.g. gene expression patterns, epigenetic imprinting, and developmental pacing). However, there is no consensus on which features should guide scientists’ predictions of developmental potential, particularly for research that aims to mimic embryonic development as closely as possible. Nor is there consensus on how heavily each feature should weigh in this assessment.

#### Benchmarks in animals

Before one can decide which features are suitable for benchmarking developmental potential in human SCBEMs, one should consider what can be used as a reference. For many stages of development, particularly post-implantation, very little data on human embryos is available. Consequently, research relies on comparison to animal models to evaluate the developmental potential of later-stage SCBEMs (Rugg-Gunn *et al.*, 2023). This builds upon the historical precedent for using animal data in cases where human experimentation is prohibited. The birth of Dolly through Somatic Cell Nuclear Transfer (SCNT), for instance, demonstrated that the technique could be applied to clone mammals (including, presumably, humans; Wilmut *et al.*, 1997). While this has never been confirmed in humans, it has been addressed in legislation (e.g. Embryos Act, 2002; Prohibition of Human Cloning for Reproduction Act, 2002). However, it is well known that animal models are not a perfect proxy for human biology. Of the commonly accepted animal models, mice post-implantation embryos are morphologically different from human (Shahbazi and Zernicka-Goetz, 2018), macaques have a longer morula stage and later implantation (9–10 days) (Nakamura *et al.*, 2016), and pig developmental speed and cell number differs (Simpson and Alberio, 2023). Thus, they are understood as not entirely representative when studying gene expression patterns, epigenetic imprints, and developmental pacing.

#### Benchmarks in human embryos

The most accessible human comparison are IVF embryos, though this reference is specific for pre- and peri-implantation models. While such embryos cannot be used for early-stage or high-throughput research, they can be useful for in-depth studies (Guo *et al.*, 2025). The quality evaluation to which IVF embryos are subject plays a significant role in determining the chance of a successful pregnancy and can, therefore, be considered predictive of developmental potential. At the same time, significant gaps persist in understanding and addressing the factors contributing to poor embryo quality, and only a minority of IVF embryos actually goes on to establish successful pregnancies. This, combined with the fact that human SCBEMs are already being used to help bridge gaps in embryo quality assessments, underscores that IVF embryos are far from ideal benchmarks.

Currently, embryo evaluation is performed from fertilization through the blastocyst stage. Evaluation of the blastocyst supports assessment of the present cell lineages: the trophectoderm (TE) and the inner cell mass (ICM) using the Gardner system (Schoolcraft *et al.*, 1999). A visual assessment by a skilled operator in the laboratory or an algorithm used in combination with timelapse imaging is performed assessing three main categories: (i) the degree of expansion and growth, (ii) the morphological appearance of the ICM, and (iii) the morphology of the TE. The importance of precise evaluation of embryo quality for reproduction is reflected in data demonstrating that the percentage of live births after single embryo transfer in ART was 54.5% in patients aged <35 years (US Centers for Disease Control and Prevention, 2020). A study by Høyer *et al.* (2021) demonstrated that following their first embryo transfer, patients had a live birth rate of 35.7%, but that this declined for subsequent transfers. These rates show that even embryos currently graded high enough for transfer do not complete development in nearly 50% of cases, indicating that many factors that play a role in a successful pregnancy outcome are likely overlooked in current evaluations. Thus, it is difficult to predict even in IVF embryos whether they truly possess developmental potential and, therefore, which embryos should serve as a reference point for SCBEMs.

Given our incomplete understanding of early-stage human development, the qualitative nature of the Gardner system may not fully encapsulate the complexities of embryogenesis (Rijnders and Jansen, 1998; Milki *et al.*, 2002; Rocha *et al.*, 2016). To overcome this, a range of methodologies has been suggested to complement the Gardner system that may also serve as benchmarks for SCBEMs developmental potential. Physiological measurements of the embryo’s cellular respiration, developmental pacing, zona pellucida birefringence index, and metabolism (Hoshi, 2003; Held *et al.*, 2012; Thompson *et al.*, 2016; Harada *et al.*, 2020), as well as novel molecular-omics technologies, such as transcriptome sequencing (Groff *et al.*, 2019), have been suggested as candidates for embryo quality assessment strategies. These measurements can be done either non-invasively (respiration, developmental pacing, birefringence index, and metabolism) or require a biopsy (transcriptome sequencing) and several studies indicate results are predictive of embryo quality (Lopes *et al.*, 2007; Madaschi *et al.*, 2009; Huang *et al.*, 2016; Giménez *et al.*, 2023).

To overcome the inter- and intra-investigator variability of visual assessment, computer-based systems and artificial intelligence may provide supportive tools (Salih *et al.*, 2023). Chen *et al.* (2016) created a computer-assisted scoring system to successfully predict embryo implantation potential in IVF/ICSI. Data-driven systems trained on embryogenesis time-lapse imaging videos have also demonstrated increased predictive accuracy compared to manual selection by embryologists, showcasing the potential of technology in improving embryo selection processes (Santos Filho *et al.*, 2012). However, as the translation of research findings into clinical implementation is a lengthy and complicated process, visual assessment by highly skilled embryologists using the Gardner system so far remains the gold standard of embryo quality assessments.

Beyond the embryo’s internal features, the embryo’s environment may either support or disrupt its development. Examples of external features are nutrients, growth factors, cytokines, and hormones present in the uterus, as well as physical and biochemical conditions, e.g. temperature and oxygen tension, and the mechanical features of the endometrium and underlying tissues (particularly during implantation) (Armant, 2005; Guzeloglu-Kayisli *et al.*, 2009; Yamanaka *et al.*, 2011; Li and Winuthayanon, 2017; Sternberg *et al.*, 2021). These features are difficult to study in humans, although progress has been made to study endometrial tissue *in vitro* (Turco *et al.*, 2017; Boretto *et al.*, 2019). Still, little remains known about embryo interactions with the maternal environment and the role of maternal tissue in guiding (post-implantation) development, leaving the question of what role these ‘external’ factors have in developmental potential. As such, the main focus in current embryo quality evaluation is on the embryo’s ‘internal’ features, despite what is considered an ‘internal’ or ‘external’ factor being amenable to change. The pivotal study by Shahbazi *et al.* (2016), for example, which would later also pave the way for the development of post-implantation SCBEMs, demonstrated that the uterine environment was not as essential for culturing embryos up to 14 days post-fertilization as previously thought. Similarly, the culture of SCBEMs like gastruloids, as well as certain studies on *ex vivo* culture of murine embryos, might be taken to suggest that the role of the maternal environment and extraembryonic tissues will become replaceable with growth factors and bioreactors (Moris *et al.*, 2020; Aguilera-Castrejon *et al*., 2021).

#### Benchmarks in human SCBEMs

Unlike human embryos, SCBEMs are more amenable to scientific experimentation and facilitate the pursuit of endpoint and high-throughput assays, yielding quantitatively comprehensive and conclusive readouts. However, in order to provide faithful alternatives in research, these readouts must be benchmarked against libraries of existing data on human embryos (de Graeff *et al.*, 2023; Martinez Arias *et al.*, 2024), including transcriptomic and epigenetic sequencing data sets (Huang *et al.*, 2022; Zhao *et al.*, 2025), before any individual SCBEM’s features can be classed as a benchmark.

To do so, the list of embryo quality assessment methodologies needs to be expanded beyond what has currently been shown to be predictive of embryo quality in IVF laboratories. Within the Gardner system, the focus lies on features such as embryo morphology and growth rate. In recent years, other features have been mapped using human embryos donated for research, such as the transcriptome and epigenome of the different cell types in the pre-implantation stages (Huang *et al.*, 2022; Zhao *et al.*, 2025). Less studied are the embryo’s metabolism and changes in energy demand (Gardner and Wale, 2013). These parameters define the pace of development and are therefore crucial for embryos to reach the right stage at the right time, e.g. blastocysts should have formed and be ready to implant during the window of implantation. Moreover, recent work in mouse SCBEMs demonstrated the role of glycolysis in directing cell fate during gastrulation (Stapornwongkul *et al.*, 2025). Thus, our understanding of the development potential of human SCBEMs is tied to the development of more predictive methods of human IVF embryo potential.

### Developmental potential in ethics

The emerging consensus that the stringency of regulation on research with SCBEMs should depend upon whether specific models can be considered to possess a developmental potential akin to human embryos underscores the historical role of the AFP. In the literature, the AFP is taken to reflect the view that early human embryos differ from other (clusters of) human cells in terms of what they can become: a human person like us, rather than one of its parts. This difference in potentialities is thought to bear moral significance, for example, making (early) human embryos intuitively more considerable than gametes or other human cells. However, the kind and the degree of this consideration remain contested (Pereira Daoud *et al.*, 2024). This is, at least in part, because the question of developmental potential is not a purely biological or empirical one (Villalba *et al.*, 2024). As observed by Lizza, ‘disagreement over ascriptions of potentiality may (…) stem from disagreement over the normative assumptions behind those ascriptions’ (Lizza, 2014a, 11). The following sections aim to help clarify how the concept of developmental potential is used by providing a taxonomy of developmental potential in terms of possibility, probability, and predisposition (Table 2).

**Table 2.** Overview of developmental potential accounts.

Account	Claim	Type of claim	Type of answer	Distinctions in potential	Conditional on	Origin of moral value	
Possibility	X can change into Y	Consequentialist (future state)	Binary (yes or no)	No: potentials are either there or they are not	External factors: contingent circumstances	Extrinsic	
Probability	X is likely to change into Y	Consequentialist (future state)	Gradual (more or less)	Yes: some potentials are more likely than others	External factors: cohort data	Extrinsic	
Predisposition	X is predisposed to change into Y	Ontological (present state)	Binary (yes or no)	Yes: some potentials are autonomous and identity-preserving (i.e. ‘active’), others are not (i.e. ‘passive’)	Internal factors: autonomous and identity-preserving development	Intrinsic	Form follows function
							Function follows form (‘threshold-views’)

This table outlines the different ways in which developmental potential can be conceptualized (‘Account’), the forms their claims take (‘Claim’), and how these claims can be evaluated (‘Type of claim’). In addition, it highlights how each account qualifies potential (‘Type of answer’), whether this qualification supports drawing distinctions between kinds of potentials (‘Distinctions in potential’), what those distinctions depend upon (‘Conditional on’), and where their respective moral value originates from (‘Origin of moral value’).

#### Possibility accounts

Those that interpret developmental potential as possibility (Tooley, 1983; Harris, 1985; Tauer, 1985; Lizza, 2014b) describe an entity’s capacity to undergo continuously organized human development as one of many possible future outcomes. For advocates of this view, to say that an embryo has developmental potential is essentially to say that ‘if certain things happen to it (like implantation), and certain other things do not happen (like spontaneous abortion), it will eventually become a human being’ (Harris, 1985). This means, first, that possibility statements about potential are binary in structure (i.e. something either is or is not possible under a particular set of circumstances) and, second, that numerous potentials are conceivable for any given thing. Consider, for example, ‘the potential of a pile of dehydrated orange juice. The powder is potentially orange juice (just add water). However, it is potentially many other things as well. All that we have to do is adjust the conditions. Since we could add water and arsenic, it is potentially poison. Since we could add flour, eggs, and yeast, and then bake, it is potentially orange cake’ (Lizza, 2014a, 10). On this view, whatever potential human embryos—and piles of dehydrated orange juice—eventually realize, is ultimately contingent upon a constellation of (arbitrary) external factors. An embryo for which the window of implantation has passed, for example, inevitably lacks developmental potential on this view because, under this particular set of circumstances (including the fact that full ectogenesis cannot presently be performed), there is no possibility of it ever developing to term. Let us call this the Possibility Account.

The fact that Possibility Accounts consider developmental potential to depend on external intervention has normative implications. As explained by Brown, developmental potential so understood can ‘entitle an entity only to the respect due the external source of its value’ (Brown, 2007), meaning that whatever protection an entity is due on this basis cannot, by itself, ground categorical prohibitions.

#### Probability accounts

Those that interpret developmental potential as probability (Langerak, 1979; Engelhardt, 1986; Persson and Savulescu, 2010) differ from Possibility Accounts in that they connect an entity’s capacity to undergo continuously organized human development to both possibility and likelihood. Advocates of this view ‘distinguish direct or proximate potentialities from indirect or remote ones (…) by appealing to criteria like causal importance, the degree or ease or difficulty of supplying the missing elements to realize the potential, or what occurs in the ‘normal course of events’’ (Lizza, 2014a, 10). As such, they often allow an entity’s developmental potential to be based upon both internal and external factors, though the later are again considered decisive for the realization of particular potentials.

*“An embryo has the potential to become a person if it has the appropriate genes, proteins, and other internal structures that, given a certain environment, enable it to develop into a person. Whether this potential is actualizable depends on whether the relevant external conditions, be they biological or social, (…) are sufficient to enable the embryo to realize its potential. As we conceive of the concept of actualizable potential, the concept of potentiality refers to states internal to the being and the concept of actualizability refers to states external to the being that together cause it to actualize its potential (…). Thus, such an embryo in a petri dish lacks actualizable potential to become a person because it is not in an environment that allows it to become one, whereas such an embryo in a normal uterus has this actualizable potential”* (Persson and Savulescu, 2010).

Interpretations of developmental potential as probability imply making a quantifiable statement about future states. These statements differ from those made by Possibility Accounts in that they are gradual, rather than binary, in structure. Let us call this the Probability Account. Like Possibility, Probability Accounts can only ground extrinsic, rather than intrinsic value (or moral status). Unlike Possibility Accounts, Probability Accounts base their assessments upon cohort data, which ultimately hinge upon what currently happens to be considered a ‘normal’ course of affairs (Feinberg, 1974; Tooley, 1983; Lizza, 2014a).

#### Predisposition accounts

In contrast to Possibility and Probability Accounts, advocates of developmental potential as predisposition interpret the capacity for continuously organized human development not as a speculative future state but as a present, actual property of the entity. As Matthen (2014) emphasizes, the mere *possibility* of a particular future state occurring is neither a necessary nor a sufficient condition for developmental potential in this sense. Buckle makes a similar point regarding *probability*: ‘the potentiality of an entity is not what it will probably become, but the power it has to become something, whether or not that becoming is probable’ (Buckle, 1990). Advocates of this view accept that entities may be prevented from realizing their potential, for instance, due to environmental conditions. A human embryo may never develop into a sentient and rational adult if it is not placed in a suitable uterine environment. However, this fact alone does not mean the embryo lacked developmental potential. As put by Eberl, ‘The form of external assistance a uterus provides is analogous to an astronaut’s spacesuit or an underwater explorer’s submarine. Each provides what the person needs to exercise her vital metabolic functions; the lack of such support does not entail that she lacks the relevant potentialities for those functions’ (Eberl, 2014). By ‘power’, ‘predisposition’, or ‘potential’, advocates of this view thus mean an entity’s ‘ontology. To speak of the potentiality of a being implies affirming something about the nature of that being, something about the kind of being it actually is: the biological facts about it cannot be but a posteriori confirmations and empirical expressions of an underlying ontological structure’ (Reichlin, 1997). As such, the only difference between human embryos and human adults (i.e. human persons) is one of maturation, rather than substance or kind (George and Lee, 2009). Let us call this the Predisposition Account.

Predisposition Accounts, rooted in Aristotelian—and, later, Thomistic—philosophy, distinguish between passive and active potentialities. Passive potentialities refer to the capacity of something to be changed or acted upon by something else. By contrast, active potentialities refer to the capacity of something to act or bring upon change by and to itself. Consider the potential of acorns, for example. Like dehydrated orange juice, acorns can have many potentials: they are ‘potential food, or humus, or whatever human ingenuity can make of it’ (Buckle, 1990). The key reason why these (passive) potentials are considered qualitatively different from the (active) potential (or, natural predisposition) acorns have to become oak trees is because the latter can be considered *identity preserving*, whereas the former cannot. In this sense, ‘the notion of potentiality is in fact a dynamic one, basically designed to account for a being’s continuity through change, so that that being’s identity is preserved while it completes and perfects its nature, acquiring those capacities and qualities which did not show themselves in its early stages, but towards which its development actually pointed’ (Reichlin, 1997). Whereas passive potentials hinge upon external events and confer, therefore, at most extrinsic value, active potentials hold independently and bear, therefore, intrinsic moral significance.

Identity preservation is thus crucial for developmental potential to qualify as active (and, therefore, as morally significant) on Predisposition Accounts: an embryo cannot be harmed or have a (stake in its future) welfare unless it is already, in some sense, ‘the same individual as the human organism at subsequent stages of development’ (George and Lee, 2009). However, the claim that zygotes and early embryos can be identical to the later individuals to which they give rise runs into challenges in the context of biological phenomena such as fission (twinning) and fusion (chimerism) (Buckle, 1990; Baldwin, 2009). During early development, embryos can divide into twins, raising doubts about whether identity can persist across such transformations. As Baldwin explains, if twinning occurs, neither twin can be identical to the original embryo; and if twinning is always a possibility, it undermines the idea that any later person is strictly identical to an early embryo—even if division never actually occurred:

*“As we imaginatively track our life back to its beginning, it might seem obvious that each of us starts off as a particular zygote—but there is a familiar problem here. During the first two weeks or so, some embryos divide to become, as we say, ‘identical’ twins. Such twins, however, are not strictly identical to each other, even if they share the same genome. Moreover, once the difference between them is recognized, it follows that neither of them can be strictly identical to the embryo whose division gave rise to them; each of them came into existence through the process of division and did not exist earlier. Equally, therefore, the embryo that divided is not ‘the same individual as the human organism at subsequent stages of development’; on the contrary, that embryo ceased to exist when it divided. Therefore, (…) as division into twins is a possibility for any early embryo, it follows that for any later human being there is a possibility that it was not strictly identical to the early embryo that gave rise to it. From this it follows, given the necessity of identity, that no later human being is in fact identical to an early embryo, even if twinning did not occur. The mere possibility of twinning is sufficient to undermine strict identity”* (Baldwin, 2009).

Not all advocates of Predisposition Accounts are persuaded by this argument (Stone, 1994; Gomez-Lobo, 2004). Stone, for example, for whom *biological continuity* is sufficient, questions this assertion on the basis that ‘There is, as yet, no reason to believe that the division of the original cell mass is genetically determined. As the original multi-celled human animal has a biological nature the actualization of which involves a great conscious good for him, he has an interest in continued life and is owed our care and protection. Indeed, (…) if we can, we owe it to him to prevent his twinning’ (Stone, 1994).

Despite disagreements about the grounds and onset of identity preservation, the fact that it is required means that, from an ontological point of view, Predisposition Accounts are inevitably binary: human embryos either are or are not the same beings as the later human adults (or persons). This ontological claim, however, does not straightforwardly settle the question of moral status. Eberl, for instance, notes that ‘if an embryo or foetus has the ontological status of a person, it is certainly arguable that it should be regarded as having the moral status of a person as well’ (Eberl and Lizza 2014). However, such an argument must be made; it is not guaranteed by ontology alone.

Some advocates of Predisposition Accounts argue that because the embryo belongs to a biological kind—the human species—whose members are self-conscious and rational beings (i.e. persons), it should be endowed with the same moral status (Gomez-Lobo, 2004; George and Lee, 2009). On these views, (full) moral status is grounded not in the immediate exercise of personhood but in a basic capacity for it. As Brown summarizes: ‘Germans, for example, have the immediately exercisable capacity to speak their native tongue, but the ability to speak German must be understood as the consequence of a more basic potential to learn language that is part of being human. The capacity for reflective rational autonomy that endows adult human beings with moral status is said to be merely the activation of a more basic potential that is possessed by human beings as such’ (Brown, 2007). Indeed, on these views, the properties associated with human adults are merely the manifestation of a more basic capacity (i.e. form follows function, Table 2), making any distinctions between human beings ultimately arbitrary. In other words, ‘The difference between a being that deserves full moral respect and a being that does not (…) cannot consist only of the fact that, while both have some feature, one has more of it than the other—one has some arbitrarily selected degree of the development of some feature or property, whereas the other does not’ (George and Lee, 2009).

Other advocates of Predisposition Accounts challenge this conclusion (Brown, 2007; Matthen, 2014). Drawing moral distinctions between human beings based on whether they do or do not manifest the properties associated with personhood need not be arbitrary, for example, insofar as human beings (e.g. early embryos) lack the material substrates required to manifest said properties (i.e. function follows form, Table 2). Brown, for example, contends that ‘actualized properties matter more, for good or ill, than the same properties when merely potential’ (Brown, 2007), and that meaningful differences exist between First and Second Order potentials. On this account, ‘Second Order Potential, like all dispositional traits, can be attributed only on the basis of an identifiable causal substrate, a physical structure of some kind, which channels changes in the individual’s internal state in the direction of an expanded capacity to respond to events’ (Brown, 2007). Matthen makes a similar argument by drawing on ‘Aristotle’s notion of a *first actuality*’ (Matthen, 2014). These ‘threshold-views’ of Predisposition Accounts may align more closely with contemporary biology and support the idea that moral status can come in degrees—even within ontologically binary frameworks. Several thresholds may be conceivable, each marking the emergence of new capacities—such as metabolism, sentience, and cognition—that were not possible before, and that can justify different levels of moral consideration.

Taken together, debates among advocates of Predisposition Accounts highlight the importance of distinguishing ontological claims from moral conclusions. The move from *what something is* to *what is morally owed to it* is not self-evident and need, therefore, not entail the particularly controversial conclusion that (early) embryos should be endowed the same moral status as full-fledged human beings.

## Discussion

The following sections build on previous insights to clarify how developmental potential can be conceptualized and assessed in SCBEMs. First, criteria are distilled from descriptions in expert recommendations to identify what is commonly considered relevant when distinguishing between types of SCBEMs. Second, scientific assays used to assess developmental potential in IVF embryos are examined to consider how they might inform—or require adaptation for—the evaluation of SCBEMs. Third, contemporary governance approaches to SCBEMs are discussed in relation to ethical interpretations of developmental potential as possibility, probability, and predisposition. By bringing together insights from governance, science, and ethics, the discussion aims to identify underlying criteria and tensions, and to support the development of coherent and transparent governance frameworks for research with SCBEMs.

### Criteria for developmental potential from governance

There are subtle but meaningful differences in how expert recommendations currently distinguish between types of SCBEMs (Table 1). Comparison of these descriptions and the differences between them suggests that it is possible to distinguish between models based on at least three criteria, namely their (complexity in terms of) composition, organization, and interaction.

Composition refers to what SCBEMs are made of. This criterion is widely shared across expert recommendations and refers primarily to the diversity in cell types, tissues, or structures—i.e. embryonic and extraembryonic components—required for SCBEMs to be considered (more) advanced. Still, views diverge on the exact composition required to distinguish between types of SCBEMs. Some groups describe more advanced SCBEMs as containing ‘all cell types required for the development of both the foetus and its supporting (extraembryonic) tissues’ (Writing Group of the ESHRE Ethics Committee *et al.*, 2024), whereas others consider it sufficient that they ‘contain the relevant’ (ISSCR, 2021) or ‘at least part of the different extraembryonic appendages (trophoblast and primitive endoderm)’ (Bruno *et al.*, 2023). Notably, however, some of these descriptions omit the temporal dimensions of cellular composition: certain cells are only present at particular points in time, and a full account of composition would need to acknowledge this.

Organization refers to the spatial and/or temporal morphology of SCBEMs: are the distinct components of particular models arranged in a biologically meaningful way? This criterion can be evidenced in, for example, the description of the Dutch Health Council, according to which (more) advanced models ‘look just like classical embryos under the microscope’ (HCN, 2023), as well as the Australian description, which distinguishes between types of SCBEMs based on whether they ‘demonstrate ‘organised development of a biological entity’’ (NHMRC, 2022).

Interaction addresses the question of whether the different (e.g. embryonic and extraembryonic) components of SCBEMs are interacting in a biologically functional way. By contrast to the previous criterion, the focus here is not (only) on how SCBEMs look but on how they behave. Foregoing descriptions underline the importance of this functionality by distinguishing between SCBEMs based on whether they have the capacity to perform a series of actions consecutively, like modelling ‘the developmental processes of the embryo’ (UK Parliament Post, 2024) and undergoing further (integrated) human development (ISSCR, 2021). Often, this functionality is specifically linked to composition (i.e. the presence of both embryonic and extra-embryonic tissues), which suggests an understanding of functionality as ‘function follows form’ rather than ‘form follows function’.

Finally, it is worth noting that some expert recommendations suggest distinguishing between SCBEMs and embryos based on the origin and intentions behind their creation. For example, both French (Bruno *et al.*, 2023) and British (NCOB, 2024) recommendations note that SCBEMs are generated from stem cells rather than the fusion of gametes, and that this difference in biological origin may carry moral significance when compared to embryos. They further observe that SCBEMs are produced explicitly for research purposes, whereas embryos are typically created for reproduction. These proposals raise questions, particularly when considering the regulation of research with SCBEMs vis-à-vis the regulation of research with embryos that were not created through fertilization methods or with reproductive intent. However, since these differences in origin and intentionality are not used to differentiate between SCBEMs *an sich*, they are not pursued further here.

### Criteria for developmental potential from science

Many of the criteria applied to determine embryo quality could also be applied to assess SCBEMs of a similar stage. The Gardner system, for example, can be applied to blastoids to grade their morphological similarity to blastocysts (Table 3). Moreover, the assessment of SCBEMs’ transcriptomes via comparison to a human embryo reference database is already a widespread method to determine whether the cells in SCBEMs are similar to the cells present in the stage they aim to mimic (Fan *et al.*, 2021; Yanagida *et al.*, 2021; Kagawa *et al.*, 2022; Karvas *et al.*, 2023; Liu *et al.*, 2023; Oldak *et al.*, 2023; Pedroza *et al.*, 2023; Weatherbee *et al.*, 2023; Yu *et al.*, 2023; Hislop *et al.*, 2024). For many cell types, also in post-implantation stages, arrays of marker genes have been identified that help confirm the presence of these cell types in SCBEMs (Petropoulos *et al.*, 2016; Xiang *et al.*, 2020; Tyser *et al.*, 2021) (Table 3).

**Table 3.** Putative features for similarity scoring of the human blastoid.

	Indication	Human embryo	Blastoid	
Oscillation (swelling/collapsing)	Na^+^ ion pumps in trophoblast	Every 2–4 h	Occurs, frequency unknown	
TE/ICM ratio	Blastocyst health	>0.45	0.5	
Size/Cavity size (diameter in µm)	Blastocyst health	150–200 µm	150–300 µm	
Respiration/metabolism	Cell health/Implantation potential	Oxygen consumption rate >0.5	unknown	
Zona pellucida	Hatching potential	Yes	No	

	**Marker**	**Embryo expression timepoint**	**Present in blastoids**	**Blastoid expression timepoint**

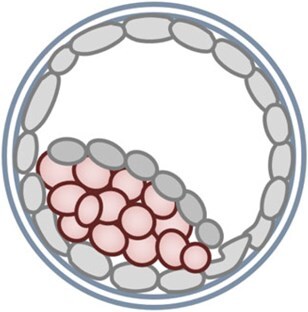	KLF17	E5–E6 (Peak at zygote E2)	Yes	Day 1
	OCT3/4	E5–E6	Yes	Day 1
	NANOG	E5–E7	Yes	Day 1
	SUSD2	E5–E7	Yes	Day 1
**Inner cell mass/epiblast**	SOX2	E5–E7	Yes	Day 1

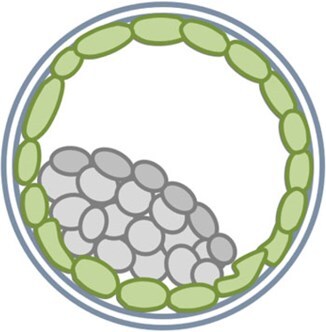	AP2 gamma	E2–E10	Yes	Day 1
	CDX2	E5–E6	Yes	Days 2–3
	GATA2	E5–E6	Yes	Day 1
	GATA3	E5–E6	Yes	Days 1–2
	NR2F2	E6–E7	Yes	Day 4
**Trophectoderm**	CK7	E8–E9	Yes	Day 4 + 4 (outgrowth)

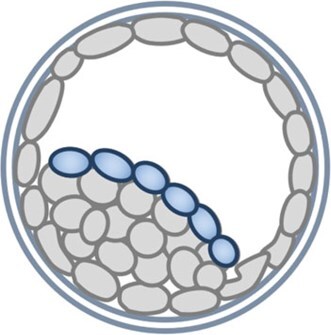	GATA6	E4–E6	Yes	Days 3–4
	HNF4a	E4–E7	Yes	Day 4
	PDGFR alpha	E5–E6	Yes	Day 4
	SOX17	E5–E7	Yes	Days 3–4
	GATA4	E6–E7	Yes	Days 3–4
**Hypoblast**	CER1	E7–E12	No	N/A

**Mesoderm**	Brachyury	CS7 (E14–E19)	No	N/A
	MESP1	CS7 (E14–E19)	No	N/A
	WNT5A	CS7 (E14–E19)	No	N/A
	CRABP2	E10–E19	Yes	Day 2.5

The upper part of the table indicates morphological and physiological features, the lower part focusses on the expression of marker genes of the three blastocyst lineages. Time points of days since fertilization (E) are indicated for the human embryo based on single-cell sequencing data in *in vitro* cultured embryos. Time points in blastoids are based on data from Yanagida *et al.* (2021) and Kagawa *et al.* (2022).

Furthermore, some models have been tested for functional features, e.g. the blastoid and its capacity for attaching to endometrium cells *in vitro* and post-implantation development (Kagawa *et al.*, 2022; Yu *et al.*, 2023). While these kinds of assays are typically not validated in human embryos, they can be indicative of developmental potential as they demonstrate the model is able to mimic developmental processes and to continue developing to a certain extent.

#### Addressing heterogeneity in SCBEMs

While several SCBEMs have a high similarity to embryos based on their morphology and transcriptome alone, major differences remain that are not (wholly) addressed by this assessment. SCBEMs can be formed from various starting cell types and these cells are usually exposed to multiple (synthetic) external signals. It remains unknown how these differences affect their capacity for further embryonic development. Moreover, SCBEMs display considerable phenotypic heterogeneity that manifests in diverse morphological outcomes, even under uniform culture conditions (Veenvliet *et al.*, 2021; Shankar *et al.*, 2024; Villaronga-Luque *et al.*, 2025). While SCBEMs are often generated in high-throughput formats, typically only a fraction of each ‘batch’ will contain the intended morphology and cellular diversity (Shankar *et al.*, 2024). This means that SCBEMs regularly include cells that do not reflect bona fide development (Zhao *et al.*, 2025). These cells are typically labelled as either ‘unclassified’ or ‘off-target’ cell types, indicating that their temporal or spatial location does not correspond to the developmental stage the SCBEMs are mimicking. The presence of these cell types may interfere with the organized development of SCBEMs and reduce their reliability as models. A key question is whether the heterogeneity of SCBEMs falls within the limits of developmental plasticity such that they can accurately recapitulate the self-organizing capacity of human embryos and their common developmental errors, such as monochorionic twinning in the blastocyst stage (Luijkx *et al.*, 2024). This question remains a critical yet unresolved issue for assessing their biological fidelity and relevance in developmental research. Moreover, the causes of the heterogeneity in SCBEM outcomes remain relatively understudied. Heterogeneity within SCBEM batches can in part be explained by two causes: (i) technical variation in culture conditions, such as different starting cell numbers between SCBEMs due to cell seeding stochasticity, and (ii) the intrinsic cellular heterogeneity in the epigenetic, transcriptomic, and metabolic states of the PSCs (Munsky *et al.*, 2012; Yang *et al.*, 2021; Villaronga-Luque *et al.*, 2025).

#### Genetic factors

Cellular heterogeneity stems in part from various kinds of genetic alterations that can arise during PSC expansion *in vitro*, from point mutations to chromosomal amplification, deletion, inversion, and translocation (The International Stem Cell Initiative *et al.*, 2007), which are currently not well-addressed in SCBEM reports. Such abnormalities, despite not impacting cell expansion, can lead to developmental disruptions or arrest when used for culturing SCBEMs, as clearly exemplified in human embryos (Shahbazi *et al.*, 2020).

#### Epigenetic factors

Another cause of heterogeneity in SCBEMs may be epigenetic alterations occurring during PSC or SCBEM culture. Increased evidence suggests that epigenetic alterations, such as in epigenetic imprinting, may limit the post-implantation potential of SCBEMs. These chemical modifications, essential for cell differentiation and development, likely differ between SCBEMs and embryos due to their distinct origins. Typically, during extended propagation of human PSCs, parental-specific DNA methylation imprints are not sustained (Guo *et al.*, 2017). Alteration or loss of these imprints (Guo *et al.*, 2017) can potentially lead to developmental malformations, as has been shown in various studies of *in vitro* cultured human embryos (Jacob and Moley, 2005).

In conclusion, while SCBEMs present promising opportunities for the study of human development, the heterogeneity of these models warrants more thorough characterization to determine the extent to which they faithfully replicate human development and, thus, merit similar regulation to the human embryos. By refining culture conditions to reduce heterogeneity and developing robust benchmarking systems that account for the remaining heterogeneity in morphology, (epi)genetics, metabolism, etc, researchers can better assess the relevance and reliability of SCBEMs.

#### Additional features for similarity scoring

**Figure 2.**
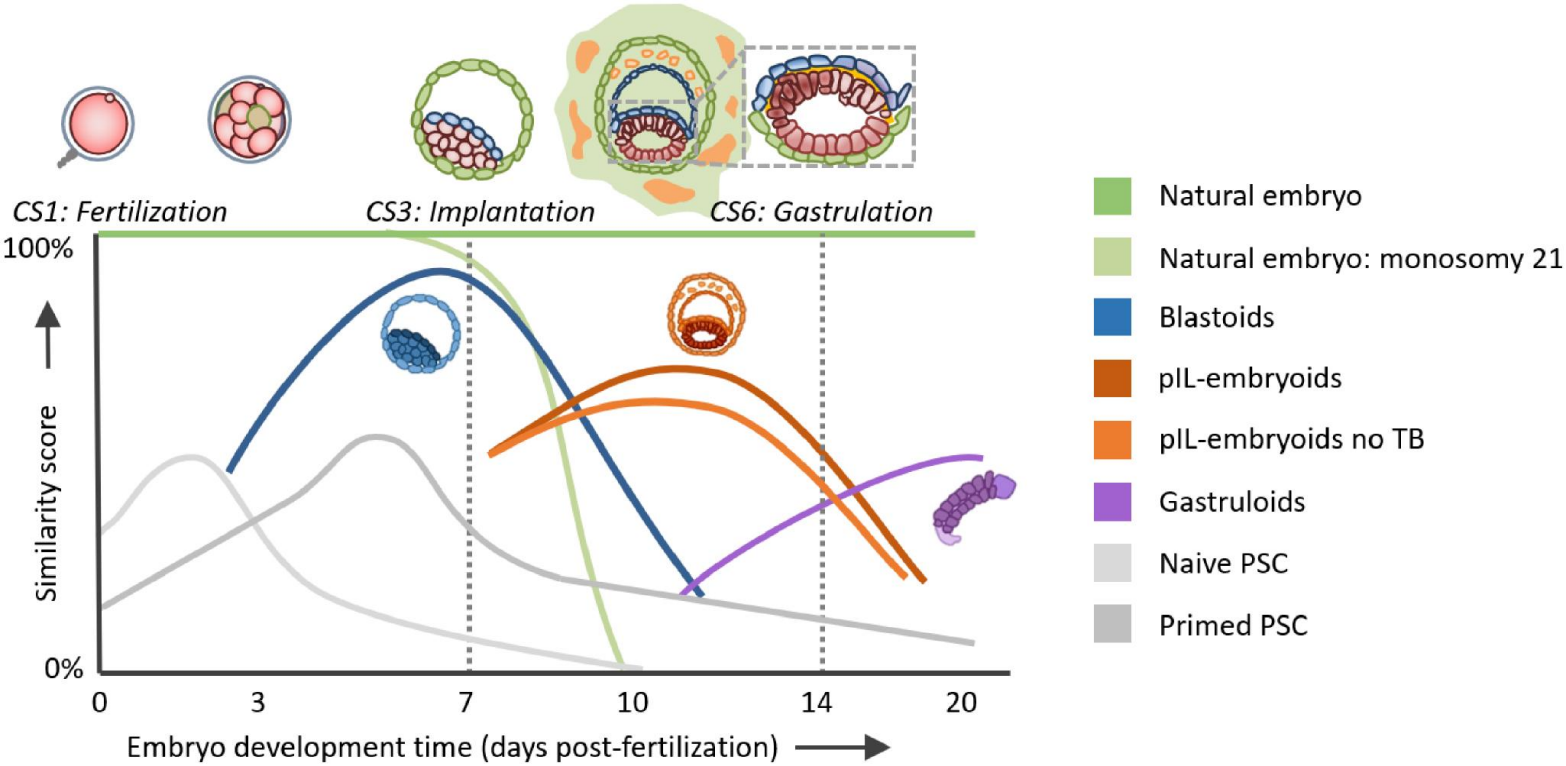
**Similarity of pluripotent stem cells (PSCs) and SCBEMs to natural embryos across key Carnegie stages (CS) of early development.** From fertilization (0 days post-fertilization) to post-gastrulation (20 days post-fertilization). Similarity scores (*y*-axis) indicate how closely each model mimics the morphology and molecular features of the natural embryo (dark green line). Natural embryos containing a non-viable (meiotic monosomy 21) aneuploidy (light green) diverge after implantation. Blastoid models (blue) exhibit high similarity around implantation but decline thereafter. Post-implantation-like embryoids (pIL-embryoids; dark orange) extend further into post-implantation stages, with reduced similarity when the extraembryonic trophoblast (TB) is missing (light orange). Gastruloids (purple) model aspects of gastrulation but lack early developmental features.

While current SCBEMs remain in early technological development stages, with an eye on the historic regulation of stem cell research and ARTs, it is imperative that researchers align on a system for benchmarking the SCBEMs’ similarity to human embryos and thus predicting their developmental potential. Through such cooperation, scientists can provide legislators with the scientific data they need to make appropriate ethical and legal recommendations, as well as reduce heterogeneity in SCBEMs and unleash their true potential for modelling early human development. Therefore, the existing assays, such as immunofluorescence for cell lineage markers and single-cell sequencing, should be complemented by the various methods when scoring SCBEMs, particularly blastoids, to benchmark their developmental potential compared to human embryos (Fig. 2).

For example, alongside the standard Gardener score assigned to three measurements—namely, blastocyst development stage, ICM, and TE quality—other morphological parameters may be used to assess blastoid quality and similar morphological assessments should, where possible, be adopted for other SCBEMs. Timelapse imaging may be applied to quantify developmental time *in vitro*, aligning this with what we know of human development timelines. The presence of oscillation in cavity sizes (Kagawa *et al.*, 2022) or patterns of expansion should also be noted, as these features suggest that the cells within SCBEMs function similarly to those in the embryo.

Physiological measurements of cell respiration and metabolism could supplement morphological quantifications. For example, cell metabolism can be analysed by infrared microscopy (Vergouw *et al.*, 2008; Venturas *et al.*, 2022). This technique could be applied to both blastocysts and blastoids, which may give insight into the differences between the two. Furthermore, whole-genome sequencing may provide understanding of the genetic background of successful SCBEMs, complimenting existing data from transcriptomic analyses such as RNA-sequencing, particularly in relation to developmental timepoints. Thorough comparative single-cell analyses allow researchers to benchmark the quality of SCBEM cell lineages across protocols, as done for blastoids by Balubaid *et al.* (2024) as well as Kim *et al.* (2024). The high-throughput nature of SCBEMs lends itself to the generation of large numbers of data points, which in turn prompts the concept of automated and/or data-driven analysis. Image-based analysis is already used in ART clinics, with modern embryoscopes used to analyse developing blastocysts prior to implantation (Mölder *et al.*, 2015). Researchers could use similar image-based analysis such as cell: cell tension modelling (Ichbiah *et al.*, 2023) to benchmark the development of SCBEMs. Existing databases such as CellphoneDB (Efremova *et al.*, 2020) could be used as a tool to interrogate transcriptomic data.

In summary, current assessments of SCBEMs require further elaboration to truly understand their developmental potential. The suggestions above are by no means exhaustive and should be expanded upon as new quality evaluation techniques and tools become available.

### Criteria for developmental potential from ethics

Several expert recommendations adopt binary descriptions for SCBEMs (Table 1). Typically, such binary approaches are characteristic of Possibility and Predisposition accounts, both of which propose clear dichotomies: the answer to the question of whether an entity has the potential to develop into a human being within a particular set of circumstances is then either yes or no (Table 2).

For Possibility accounts, whether (or not) this potential exists is ultimately contingent on external factors (Table 2), including environmental and social conditions, such as transfer to a conducive *in vivo* or *in vitro* environment. Advisory reports that describe SCBEMs in binary terms while emphasizing such external conditions could thus be interpreted as drawing on elements of Possibility accounts. The Swedish report, for example, suggests that an entity’s developmental potential depends on the timing of transfer, stating that ‘Inducing an embryo to develop into a new individual is not possible today once the window of time for implantation in a uterus has been passed. (…) The question of the embryo’s survival and the possibility of it developing into a new individual is thus not relevant to research on embryos *in vitro*’ (SMER, 2024). The French report highlights reproductive intent, noting that ‘the status of the embryo depends on the parental project: the embryo is considered «a person in the making» as long as it is part of such a project’ (Bruno *et al.*, 2023) but that this is no longer the case once it is donated for research. The Dutch Health Council’s report emphasizes societal meaning, stating that ‘In order to qualify for protection under the Embryo Act, the committee believes it should concern entities that have the capacity to develop into a human being. In this case, [this capacity] is not about the potentiality argument for moral status, but rather about a characteristic that is regarded in society as morally relevant’ (HCN, 2023).

For Predisposition accounts, external factors are neither necessary nor sufficient conditions for developmental potential (Table 2). Advisory reports that describe SCBEMs in binary terms *without* any reference to external factors (like transfer to a uterus or extended culture *in vitro*) could thus be interpreted as aligning more closely with Predisposition Accounts. Australia, for example, seems to focus exclusively on internal aspects, like the ability of SCBEMs to demonstrate the ‘organized development of a biological entity’ (NHMRC, 2022). Notably, this organized development is considered possible ‘even if [a particular SCBEM] does not: follow the normal embryonic development process, or undergo epithelial-to-mesenchymal transition (EMT) and/or form a primitive streak, or pass through the stage of gastrulation’ (NHMRC, 2022), which points towards an understanding of developmental potential in terms of ‘form follows function’. Another example can be found in the recommendations of the Writing Group of the ESHRE Ethics Committee. Like Predisposition Accounts, these recommendations seem to frame developmental potential as a ground for *intrinsic* moral value: ‘The moral status attributed to a human embryo determines our ethical obligations towards it, sets boundaries on our actions involving embryos, and specifies the level of protection it warrants’ (Writing Group of the ESHRE Ethics Committee *et al.*, 2024). This status is considered to be initially low and to increase step by step with the achievement of certain developmental milestones, making it compatible with Threshold Predisposition Accounts, according to which an entity’s developmental potential is preceded by the establishment of certain biological substrates (i.e. ‘function follows form’).

In contrast to Possibility and Predisposition Accounts, Probability Accounts tend to reject rigid dichotomies in favour of gradual evaluations (Table 2). Expert recommendations that avoid binary descriptions and favour case-by-case evaluations could thus be interpreted as containing elements of Probability Accounts. The report of the Nuffield Council on Bioethics, for example, states that ‘the potential of SCBEMs to develop is both varied and variable (…). It is varied in that most SCBEMs model only elements of the embryo, yet some models are more complex and complete. It is variable in that embryo models are modular and easy to adapt. There are ways in which science can limit or prevent this potential, for example, by excluding features from the model that are necessary for onward development’ (NCOB, 2024). While this statement could be considered compatible with Predisposition accounts if the ‘features considered necessary for onward development’ consisted exclusively of internal factors, this is not made explicit.

This analysis does not and cannot claim that particular advisory reports endorse particular accounts of developmental potential to differentiate between SCBEMs. Instead, it has aimed to explore whether and how the language and criteria used might connote (elements of) different accounts. Identifying which account of developmental potential is operative in a governance framework not only helps clarify the reasoning behind its specific recommendations but also reveals potential tensions or inconsistencies among them. For example, should the degree of oversight due to SCBEMs (partly) depend on external factors, like what is technically possible today? And, if so, how stable are these grounds as technologies evolve? Can bans based on Possibility or Probability Accounts ultimately sustain the normative weight they are made to carry? And if not, might Predisposition Accounts offer reasonable and biologically grounded alternatives, or are they rightfully dismissed as metaphysical?

## Conclusion

The aim of this narrative review has been to address a pressing question at the intersection of science, ethics, and governance: how should we define and assess the developmental potential of human SCBEMs? Experts recommend that we rely on the presumed capacity of SCBEMs to continuously undergo organized human development to determine the degree of oversight due to particular types of models. This capacity also plays a prominent role in ethical debates about moral consideration. Currently, however, there is still no clear or consistent way to assess it.

In the background, we showed that different expert recommendations emphasize different aspects when describing developmental potential in SCBEMs. While these differences can be subtle, they are practically significant. We also showed that the biological basis for assessing developmental potential (in embryos, let alone SCBEMs) remains poorly understood. Scientifically, developmental potential is multifaceted, and assessing it in SCBEMs will require measuring a broader range of aspects, ideally through comparisons with human embryos when possible. The range and significance of these aspects, however, is often shaped by underlying ethical interpretations of developmental potential (as possibility, probability, or predisposition), each with varying normative implications. Possibility and Probability Accounts ground extrinsic value, as developmental potential is only considered realizable upon external intervention. By contrast, Predisposition Accounts ground intrinsic value by interpreting developmental potential as an ontological claim about identity-preservation. However, the move from this ontological claim to a moral one is not self-evident, and different arguments can be made with regard to the degree of intrinsic value (full or gradually increasing) it confers.

In the discussion, we proposed an initial synthesis of these insights to clarify how developmental potential is and can be applied. We identified three common criteria used across expert reports, namely composition, organization, and interaction. Next, we considered how existing scientific assays (such as those used in IVF) might be adapted to SCBEMs. Finally, we explored whether and how current governance frameworks connote (elements of) distinct ethical interpretations of developmental potential. In doing so, our aim was not to assert that particular advisory reports endorse particular accounts, but to identify criteria and tensions in how the concept of developmental potential is connoted, and to equip stakeholders—including regulators, scientists, and ethicists—with the tools and shared language needed to move contemporary classifications of SCBEMs forward.

Our findings raise important questions for future research. For example, if developmental potential is considered intrinsically valuable (like on Predisposition Accounts), what biological markers could be considered to indicate autonomous and identity-preserving development? By contrast, if the value of developmental potential is extrinsic (like on Possibility or Probability Accounts), what kinds of evidence might be needed to justify categorical limits or bans? Questions also arise from a scientific perspective. Is there an ideal benchmark for SCBEMs? And which aspects should count most when making comparisons between SCBEMs and embryos? Answering these questions will require meaningful and ongoing collaboration across disciplines. Ultimately, our analysis highlights that debates about human SCBEM research hinge on how developmental potential is conceptualized, assessed, and valued. By clarifying the different ways in which it can be understood and ground moral and regulatory consideration, we hope this review contributes to the responsible development and governance of SCBEM research.

## Supplementary Material

dmaf033_Supplementary_Data

## Data Availability

No new data were generated or analysed in support of this research.
