# Supplementary material for: Beyond integration: towards benchmarks for developmental potential in human stem cell-derived embryo models
Source: Hum Reprod Update. 2026 Jan 5;32(3):313–32. doi: 10.1093/humupd/dmaf033 (PMC13139666; doi:10.1093/humupd/dmaf033)
Supplement: dmaf033_Supplementary_Data [file dmaf033_supplementary_data.pdf]

Title: Beyond Integration: Towards Benchmarks for Developmental Potential in Human Stem Cell-Derived Embryo Models

Running title: Beyond Integration in Human Stem Cell-Based Embryo Models

Authors: Dorian G. LUIJKX[1,†] (ORCID: 0000-0001-8670-1470), Leila ASHTAR[1,†] (ORCID: 0009-0001-0513-8549), Nienke DE GRAEFF[2,3] (ORCID: 0000-0002-0045-4366), Edith COONEN[4,5,6] (ORCID: 0000-0002-8601-7369), Stefan GISELBRECHT[1] (ORCID: 0000-0002-1354-0167), Guido M.W.R. DE WERT[5,7,8] (ORCID: 0000-0002-0410-4902), Erik J. VRIJ[1,5] (ORCID: 0000-0003-0702-5609), Rhiannon GRANT[9] (ORCID: 0000-0003-1507-2042), Ana M. PEREIRA DAOUD[2,*] (ORCID: 0000-0002-0675-8133).

[1]MERLN Institute for Technology-Inspired Regenerative Medicine, Department for Cell Biology-Inspired Tissue Engineering, Maastricht University, Maastricht, The Netherlands.

[2]Department of Medical Ethics and Health Law, Leiden University Medical Center, Leiden, The Netherlands.

[3]The Novo Nordisk Foundation Center for Stem Cell Medicine (reNEW), Leiden Node, Leiden, The Netherlands.

[4]Department of Clinical Genetics, Maastricht University Medical Center, the Netherlands.

[5]Department of Obstetrics & Gynecology, GROW Research Institute for Oncology and Reproduction, Maastricht University, Maastricht, The Netherlands.

[6]Department of Reproductive Medicine, Maastricht University Medical Center+, the Netherlands.

[7]Department of Health, Ethics and Society, Maastricht University, Maastricht, The Netherlands.

[8]Care and Public Health Research Institute (CAPHRI), Maastricht University, Maastricht, The Netherlands.

[9]Institute for Bioengineering, School of Engineering, University of Edinburgh, Scotland, United Kingdom

†These authors contributed equally.

***Corresponding author:** Albinusdreef 2, 2333 ZG Leiden, the Netherlands. E-mail address: a.m.pereira_daoud@lumc.nl (ORCID: 0000-0002-0675-8133)

Table of Contents

Supplementary Data File S1: Glossary 2

Supplementary Data File S1: Glossary

Axis development: Establishment of anterior–posterior, dorsal–ventral body axes during early embryogenesis.

Birefringence index: An optical property of a material with a reflective index that depends on the polarization direction of the light.

Blastocyst: The preimplantation stage embryo consisting of an inner cell mass, trophectoderm, and blastocoel cavity, typically formed 5–6 days post-fertilization.

- **—Trophectoderm (TE):** The outer epithelial layer of the human blastocyst that gives rise to the extraembryonic tissues of the placenta and mediates implantation into the maternal endometrium.
  - **—Extraembryonic tissues:** Tissues that will not constitute the body of the developing organism, but which support its development by providing the necessary nutrients and protective environment.
- **—Inner Cell Mass (ICM):** A pluripotent population of cells taking place inside the human blastocyst, that will give rise to the epiblast and hypoblast.
  - **—Hypoblast:** A group of cells overlying the epiblast that give rise to the yolk sac during embryonic development.
    - **—Yolk sac:** An extraembryonic membranous structure providing nutrients and functioning as the initial site for haematopoiesis and germ cell development in the developing embryo.
  - **—Epiblast:** The pluripotent cell compartment of the human blastocyst that gives rise to the embryonic tissues.
    - **—Embryonic tissues:** Tissues that will constitute the body of the developing organism.

Epigenome: The collection of all chemical modifications of DNA and histones in a cell or organism that control gene expression.

—Epigenetic imprinting: An epigenetic process in which one copy of the genetic material from either maternal or paternal origins is silenced.

Gastrulation: The process through which the three germ layers are formed from the epiblast.

—Germ layers: The three primary layers ectoderm, mesoderm, and endoderm formed during gastrulation, giving rise to all tissues and organs.

—Ectoderm: The outer layer of the primary germ layers, ectoderm derivatives will give rise to epidermis, central and peripheral nervous systems, and the neural crest cells.

—Mesoderm: The middle layer of the primary germ layers, mesoderm derivatives will give rise to muscular tissues (skeletal, smooth and cardiac), skeletal tissues (bone, cartilage) connective tissue (dermis, stroma) and blood vessels.

—Endoderm: The inner layer of the primary germ layers, endoderm derivatives will give rise to epithelial lining of the digestive and respiratory tracts.

Gene expression patterns: Configurations of sets of genes that are either turned off or on within a cell. The level of gene activity determines the production of gene products, e.g., RNAs and proteins. These products, in turn, define a cell's function, its response to environmental cues, and/or its future development.

Morula: A solid mass of cells (8-32 cells) that forms after several rounds of cleavage post-fertilization.

Stem cells: Cells that can self-renew and continuously reproduce themselves, and that can also differentiate into daughter cells with different features.

—Pluripotent Stem Cells (PSCs): Cells that can self-renew indefinitely and differentiate into any specialized cell type of the body.

—Naive: PSCs that resemble cells found in preimplantation stage embryos, from the late morula to early blastocyst stage.

—**Primed:** PSCs that resemble epiblast cells found in early post-implantation stage embryos, prior to gastrulation.

Stem cell-based embryo models (SCBEMs): 3D *in vitro* structures composed of stem cells that resemble embryonic and/or extraembryonic tissues and their developmental processes.

—**Complete vs. incomplete:** Dichotomy in regulatory terminology to distinguish between types of SCBEMs, as introduced by Dutch experts. The hyponym **'complete'** refers to SCBEMs "in which the aim is to mimic the organized development of an intact human embryo" (Dondorp et al. 2021, 196). The hyponym **'incomplete'** refers to SCBEMs in which the aim is not to mimic the organized development of an intact human embryo (cf. Dondorp et al. 2021, 196). See also **Table 1**.

—**Intact vs. non-intact:** Dichotomy in regulatory terminology to distinguish between types of SCBEMs, as introduced by Australian and Dutch experts. The hyponym **'intact'** refers to SCBEMs that are considered equivalent to embryos. The hyponym **'non-intact'** refers to SCBEMs that are not considered equivalent to embryos. See also **Table 1.**

—**Integrated vs. non-integrated**: Dichotomy in regulatory terminology to distinguish between types of SCBEMs, as introduced by the 2021 ISSCR Guidelines. The hyponym **'integrated'** refers to SCBEMs that "contain the relevant embryonic and extra-embryonic structures and could potentially achieve the complexity where they might realistically manifest the ability to undergo further integrated development if cultured for additional time in vitro" (ISSCR 2021, 64). The hyponym **'non-integrated'** refers to SCBEMs that "experimentally recapitulate some, but not all aspects of the per-implantation embryo, for example differentiation of the embryonic sac or embryonic disc in the absence of extraembryonic cells" (ISSCR 2021, 64). See also **Table 1.** While these hyponyms have been abandoned in the revised Guidelines of the ISSCR (2025), due to their

widespread use in the literature, they are used in the narrative review to distinguish between types of SCBEMs when needed.

—Axioloids: Technical term to denote SCBEMs that mimic axial development and somitogenesis processes. These models lack extra-embryonic cells.

—Blastoids: Technical term to denote SCBEMs that mimic pre-implantation stages of the blastocyst. These SCBEMs contain all the embryonic and extraembryonic cells found in the blastocyst.

—Extra-embryoids: Technical term to denote SCBEMs that mimic post-implantation stages of roughly day 9 to 14 post-fertilization. These SCBEMs contain both embryonic and extraembryonic tissues, namely the epiblast, hypoblast, and their derivatives.

—Gastruloids: Technical term to denote SCBEMs that mimic the process of gastrulation and axis formation. These SCBEMs lack extra-embryonic cells.

—Post-implantation Amniotic Sac Embryoids (PASE): Technical term to denote SCBEMs that recapitulate the amniotic sac formation during the post-implantation phase of human embryogenesis.

Primitive streak: A temporary elongated cluster of cells in amniotes that serves as the gastrulation centre, and establishes anterior–posterior axis of the developing embryo.

Somatic Cell Nuclear Transfer (SCNT): A procedure in which the nucleus of a somatic cell is transplanted into an enucleated oocyte.

Somitogenesis: The process of forming the somites, the precursors of the vertebrae and skeletal muscle.

Transcriptome: The collection of all the RNA transcripts including coding or non-coding RNAs is cell or an organism.

—Sequencing: A high throughput RNA sequencing technique to profile the complete set of RNA transcripts within a cell or a tissue of interest.
